# Can YouTube be used as a credible source of information for COVID-19 vaccination in Italy? Angela Ancona

**DOI:** 10.1093/eurpub/ckac131.187

**Published:** 2022-10-25

**Authors:** A Ancona, M Godoy, P Bertuccio, L Gentile, A Odone, C Signorelli

**Affiliations:** 1 School of Public Health, University Vita-Salute San Raffaele, Milan, Italy; 2 Department of Public Health, University of Pavia, Pavia, Italy

## Abstract

**Background:**

The COVID-19 pandemic led to an ‘infodemic', as defined by the WHO, which made it difficult to be accurately informed on public health topics. For this purpose, many people use social media as a source of information, mainly YouTube. Given the great resonance of this platform, our study aims at assessing quality and reliability of its content regarding the COVID-19 vaccination.

**Methods:**

During March 2022, six searches were performed on the Italian YouTube platform using the following terms: “Covid vaccination”, “Covid vaccine”, “Coronavirus vaccination”, “Coronavirus vaccine”, “Sars-Cov-2 vaccination” and “Sars-Cov-2 vaccine”. A total of 329 videos were analysed, after removing 271 duplicated videos, and classified in seven types of channel. The reliability of the content was evaluated through the HoNCode score, while quality was tested using the validated DISCERN tool.

**Results:**

The most frequent category was ‘Internet Media’ (33%), while the less frequent one was ‘Educational Medical’ (7%). The content reliability (i.e. HoNCode score) resulted higher for videos produced by medical healthcare workers than non-medical ones. Concerning the quality, the DISCERN score resulted significantly higher for the Educational channels (median 46.0 for medical and 41.3 non-medical ones) as compared to Internet Media (26.5) and New Agencies (24.3).

**Conclusions:**

Although YouTube has implemented a policy against misinformation related to the COVID-19 vaccination, the study highlights that there is extreme heterogeneity in reliability and quality of videos. Content produced by non-medical users, especially “Internet Media” and “News Agencies” categories should be evaluated with attention by users, as their quality is not appropriate to the importance of the topic.

**Key messages:**

• Because of to the heterogeneity of its content, YouTube should be evaluated carefully when used as a source of information for Covid-19 vaccination.

• Content produced by non-medical users, is generally of poor quality, not appropriate to the importance of the topic.

